# Profiling analysis of long non-coding RNAs in early postnatal mouse hearts

**DOI:** 10.1038/srep43485

**Published:** 2017-03-07

**Authors:** Xiongshan Sun, Qi Han, Hongqin Luo, Xiaodong Pan, Yan Ji, Yao Yang, Hanying Chen, Fangjie Wang, Wenjing Lai, Xiao Guan, Qi Zhang, Yuan Tang, Jianhong Chu, Jianhua Yu, Weinian Shou, Youcai Deng, Xiaohui Li

**Affiliations:** 1 Institute of Materia Medica, College of Pharmacy, Third Military Medical University, Chongqing, China; 2 Center of Translational Medicine, College of Pharmacy, Third Military Medical University, Chongqing, China; 3 Riley Heart Research Center, Wells Center for Pediatric Research, Indiana University School of Medicine, Indianapolis, IN, USA; 4 Division of Hematology, Department of Internal Medicine, The Ohio State University, Columbus, Ohio, USA; 5 Suzhou Institute of Blood and Marrow Transplantation, Soochow University, Suzhou, China

## Abstract

Mammalian cardiomyocytes undergo a critical hyperplastic-to-hypertrophic growth transition at early postnatal age, which is important in establishing normal physiological function of postnatal hearts. In the current study, we intended to explore the role of long non-coding (lnc) RNAs in this transitional stage. We analyzed lncRNA expression profiles in mouse hearts at postnatal day (P) 1, P7 and P28 *via* microarray. We identified 1,146 differentially expressed lncRNAs with more than 2.0-fold change when compared the expression profiles of P1 to P7, P1 to P28, and P7 to P28. The neighboring genes of these differentially expressed lncRNAs were mainly involved in DNA replication-associated biological processes. We were particularly interested in one novel cardiac-enriched lncRNA, ENSMUST00000117266, whose expression was dramatically down-regulated from P1 to P28 and was also sensitive to hypoxia, paraquat, and myocardial infarction. Knockdown ENSMUST00000117266 led to a significant increase of neonatal mouse cardiomyocytes in G0/G1 phase and reduction in G2/M phase, suggesting that ENSMUST00000117266 is involved in regulating cardiomyocyte proliferative activity and is likely associated with hyperplastic-to-hypertrophic growth transition. In conclusion, our data have identified a large group of lncRNAs presented in the early postnatal mouse heart. Some of these lncRNAs may have important functions in cardiac hyperplastic-to-hypertrophic growth transition.

Promoting cardiomyocyte regenerative potential has been one of recent interests for combating heart failure. Numerous recent studies have shown that, in mammalian hearts, cardiomyocytes have a higher rate of proliferation to meet the demand of cardiac growth at mid-gestation. They gradually lose their proliferative activities towards late-gestation, and nearly completely withdraw from cell cycle after the first week post parturition. Simultaneously, the early postnatal hearts switch to hypertrophic growth^1^,^2^,^3^. This phenomenon is also consistent with a recent study that 1-day old neonatal mouse hearts remain capability to recover from partial ventricular apex resection or myocardial ischemia, while this potential is lost after postnatal day (P) 7, likely due to the loss of cardiomyocyte proliferative activity by P7^4^,^5^. Unraveling the underlying mechanism will provide great insight of cardiac transition from hyperplastic growth to hypertrophic growth, which will likely help to develop novel regenerative therapies for heart failure.

Micro (mi) RNAs, such as miR-195 and miR-17-92, have important roles in neonatal heart development *via* their regulation of checkpoint kinase 1 (Chek1) and phosphatase and tensin (PTEN), respectively, which are critical in regulating cardiomyocyte cell cycle activity^6^,^7^,^8^. This earlier work promoted us to explore whether other regulatory RNAs, such as long non-coding (lnc) RNAs, were also involved in neonatal heart development. LncRNAs exert various biological effects, participating in alternative splicing, gene imprinting, gene transcription, cell cycle and apoptosis, and other key biological processes^9^. Many lncRNAs are expressed in a unique spatial and temporal pattern during development, indicating that they are highly relevant to the regulation of developmental processes^10^,^11^. So far, several lncRNAs have been identified as functional regulators of cardiac development and pathogenesis of heart failure and cardiomyopathies^12^,^13^,^14^,^15^,^16^,^17^,^18^. However, whether these lncRNAs are associated with hyperplastic-to-hypertrophic growth transition is largely unknown.

In the present study, we performed profiling analyses on lncRNA expression in early postnatal hearts using Mouse LncRNA Microarray v2.0 Service from Arraystar, Inc (Agilent Technologies). We identified over thousands of lncRNAs *via* bioinfomatic analysis that were differentially expressed from P1 to P28. We then validated 10 lncRNAs by qRT-PCR for their expression levels and tissue specificities. Among them, we focused on one novel cardiac-enriched lncRNA, ENSMUST00000117266, which was likely involved in the hyperplastic-to-hypertrophic growth transition *via* its role in regulating cardiomyocyte proliferation.

## Results

### LncRNA profiling in early postnatal murine heart

**Figure 1:**
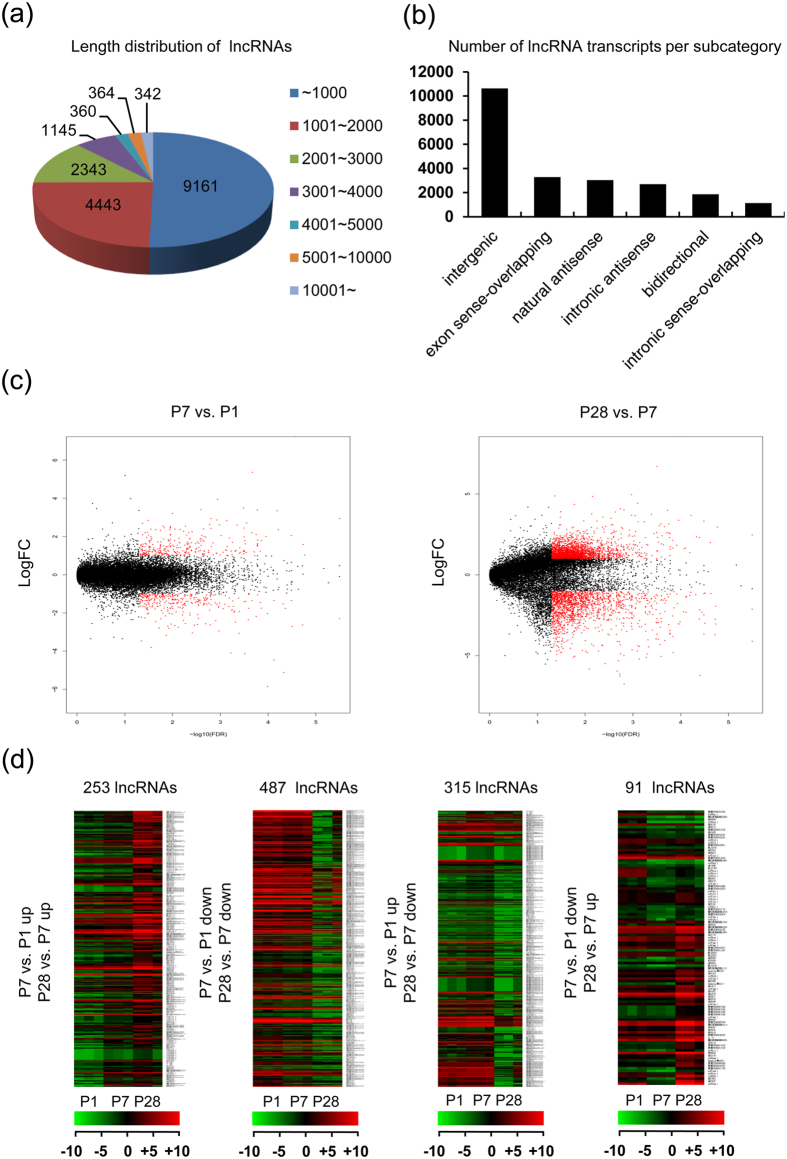
Global description of lncRNAs expressed in neonatal mouse heart. 18,158 lncRNAs were detected by using microarray technique. (**a**) The pie chart shows the length distribution of lncRNAs. (**b**) The Number of lncRNA transcripts per subcategory sorted by their positions relative to nearby coding genes. (**c**) Volcano plot of fold changes and corresponding FDR values for each lncRNA after comparison of P7 and P1 heart (left panel) or P28 and P7 heart (right panel), respectively. The red points represent lncRNAs with fold change ≥2.0 and FDR < 0.05. The black points represent lncRNAs with fold change <2.0 or FDR ≥ 0.05. (**d**) Heat maps show the hierarchical clustering analysis of the 1,146 differentially expressed lncRNAs with four different expression patterns. Each row represents the same lncRNA and each column represents the same sample. The expression intensity of each lncRNA in one sample is represented in shade of red or green, indicating its expression level above or below the median expression intensity across all samples, respectively. One-way ANOVA for (**c** and **d)**. (n = 3 samples per time point).

To identify the differentially expressed lncRNAs in early postnatal hearts, lncRNA microarray was performed by using C57BL/6 J mouse hearts harvested at P1, P7 and P28. In total, the signals of 18,158 lncRNAs from authoritative data sources from University of California Santa Cruz, RefSeq, Ensembl and other related literatures were captured in lncRNA microarray, with most of their length between 200 to 3000 nucleotides (Fig. 1a and Supplementary Table S1). These lncRNAs included intergenic, exon sense-overlapping, natural antisense, intronic-overlapping, bidirectional and intronic sense-overlapping transcripts, among which intergenic lncRNAs were over 10,000 (Fig. 1b). A total of 765 or 4,855 lncRNAs were differentially expressed with more than 2.0-fold change (false discovery rate (FDR) <0.05) between P1 and P7 or between P7 and P28, respectively, as shown by volcano plot analysis (Fig. 1c and Supplementary Table S2). After quantile normalization of the raw data for these three time points, any lncRNA with more than 2.0-fold change between any two groups was counted. Totally, 1,146 lncRNAs were found to meet the criteria (FDR < 0.05). Among these 1,146 differentially expressed transcripts, 253 lncRNAs were gradually upregulated, while 487 lncRNAs were gradually downregulated. As compared to P1 mouse heart, 315 lncRNAs were upregulated at P7 and then downregulated at P28; while only 91 lncRNAs were downregulated at P7 and then upregulated at P28 (Fig. 1d). Of note, when the fold change between P1 and P28 was set to over 10.0, there were still 107 downregulated lncRNAs and 46 upregulated lncRNAs (Supplementary Table S3).

### The Series Test of Cluster (STC) of lncRNA and mRNA expression profiles

**Figure 2:**
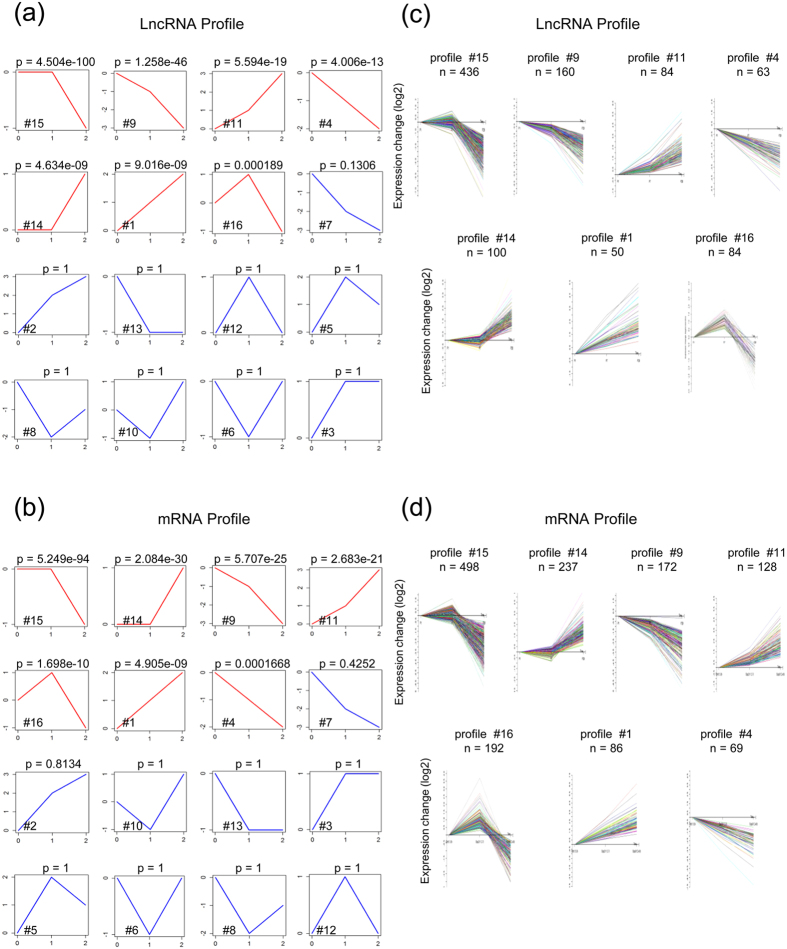
STC analysis of differentially expressed lncRNAs and mRNAs in microarray. **(a**,**b**) Sixteen model profiles were utilized to analyze the expression patterns of 1,146 differentially expressed lncRNA transcripts (**a**) and 1,358 differentially expressed coding genes (**b)**, respectively. Each box represents a different model expression profile. The *p* value and corresponding model profile number were listed in upper and lower corner of each profile box, respectively. Red or blue boxes represent model expression profile with *p* < 0.05 or with *p* ≥ 0.05, respectively. The horizontal and vertical axes represent time points and gene expression levels after Log normalized transformation, respectively. (**c**,**d**) 7 model expression profiles of lncRNA transcripts (**c**) and coding genes (**d**) with statistically significant (*p* < 0.05) are shown, respectively. Model expression profile number and the number of genes assigned to its corresponding profile are shown on the top of each profile. The horizontal and vertical axes represent indicated time points and the time series of gene expression levels after Log normalized transformation, respectively. Fisher exact test for (**a** and **b)**.

To further characterize lncRNA and mRNA expression profiles, we set up 16 expression model profiles for the 1,146 differentially expressed lncRNAs and 1,358 mRNAs in microarray by using STC method in STEM software. Each profile represents an expression model containing a group of lncRNAs or mRNAs with a similar expression pattern, respectively. 7 expression patterns of lncRNA and 7 expression patterns of mRNA showed statistically significance, respectively (*p* < 0.05) (Fig. 2a,b and Supplementary Table S4). Surprisingly, all of the 7 significant lncRNA expression patterns also existed in the significant mRNA expression profiles. We next analyzed the total number of lncRNAs or mRNAs in each significant expression pattern, respectively. Interestingly, we found that 436 lncRNAs and 498 mRNAs were assigned to Profile #15, which is the most significant pattern in both lncRNA and mRNA profile (Fig. 2c,d, top left panel). The expression of lncRNAs in Profile #15 remained temporally stable from P1 to P7 but rapidly declined at P28. This group of lncRNAs might be involved in the early postnatal heart development, as this period represents a process that the capacity of cardiomyocyte proliferation soon disappears. We also found more similar assigned number of both lncRNAs and mRNAs in the same pattern, such as Profile #9, 11, 14 and 4. The closer similarity between lncRNA and mRNA expression profiles suggested that lncRNAs might be tightly involved in mRNA expression in the early postnatal heart.

### Hierarchical clustering of differentially expressed lncRNAs and neighboring gene mRNAs

**Figure 3:**
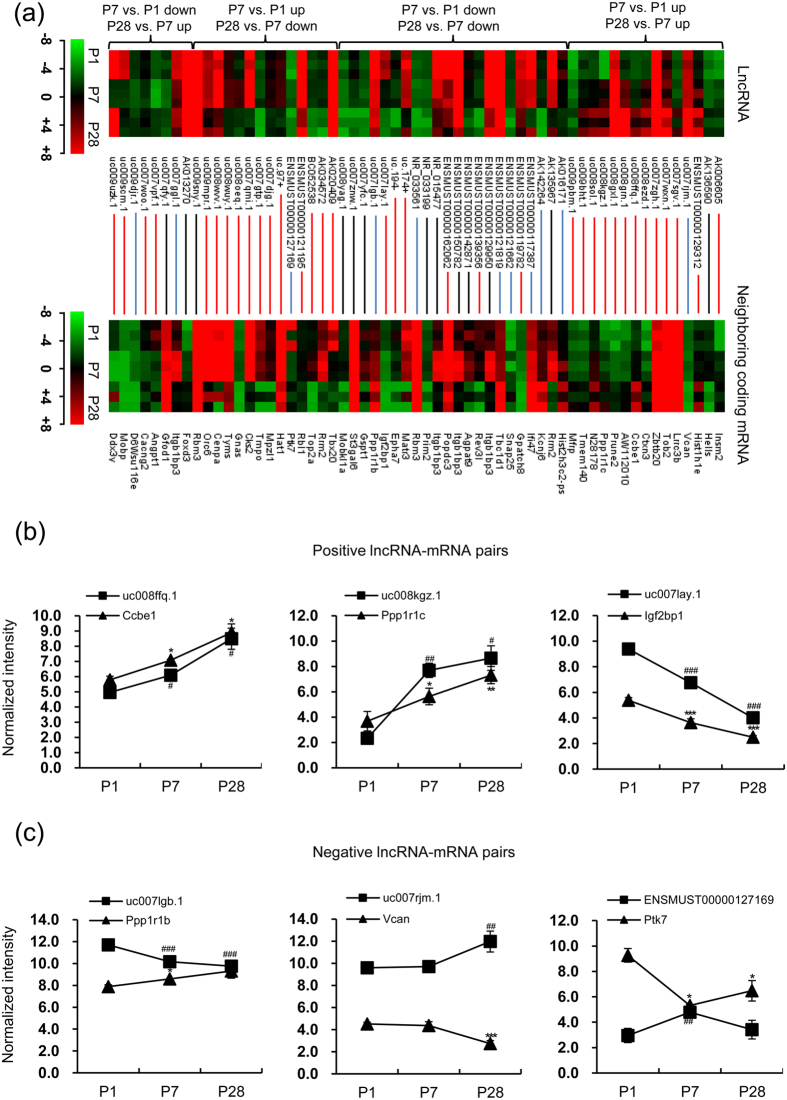
The expression levels of lncRNAs and neighboring gene mRNAs within 300 kb. (**a**) Heat maps show the expression levels of 59 differentially expressed lncRNAs (upper panel) and their corresponding differentially expressed neighboring gene mRNAs (lower panel) at three indicated time series. Each row represents one lncRNA (top) or one mRNA (bottom), respectively, and each column represents one sample. Each line indicates a lncRNA-mRNA pair. A red line represents a positive associated lncRNA-mRNA pair and a blue line represents a negative associated lncRNA-mRNA pair, respectively, while a black line means no obvious expression relevance between a lncRNA and its neighboring coding gene. The expression value of each lncRNA in one sample is represented in shade of red or green, indicating its expression level above or below the median expression value across all samples, respectively. (**b**) The normalized intensity of uc008ffq.1-*Ccbe1*, uc008kgz.1-*Ppp1r1c* and uc007lay.1-*Igf2bp1* in the microarray data. (**c**) The normalized intensity of uc007lgb.1-*Ppp1r1b*, uc007rjm.1-*Vcan* and ENSMUST00000127169-*Ptk7* in the microarray data. ^#^*p* < 0.05, ^##^*p* < 0.01 and ^###^*p* < 0.001 vs. P1 for lncRNAs. *p < 0.05, **p < 0.01 and ***p < 0.001 vs. P1 for mRNAs. One-way ANOVA for (**b** and **c)**. (n = 3 samples per time point).

It has been anticipated that lncRNA prefers to be located at the neighboring coding genes and regulates the expression of these genes^19^,^20^,^21^. Totally, we identified 879 pairs of lncRNA-neighboring coding gene partners (Supplementary Table S5), 59 of which showed differential expression in both lncRNAs and their neighboring coding genes (Fig. 3a and Supplementary Table S6). We also listed the positive and negative lncRNA-mRNA pairs whose coding genes were reported to be involved in cardiac development and function (Fig. 3b,c). For example, uc008ffq.1 along with its neighboring gene Collagen and calcium-binding EGF-like domain 1 (*Ccbe1*) and uc008kgz.1 along with its neighboring gene Protein phosphatase 1, regulatory (inhibitor) subunit 1C (*Ppp1r1c*)^22^ were consistently and gradually upregulated from P1 to P28 (Fig. 3b, left and middle panel). In addition, uc007lay.1 and its neighboring gene *Igf2* mRNA-binding protein 1 (*Igf2bp1*)^23^ were both gradually downregulated from P1 to P28 (Fig. 3b, right panel). The similar expression pattern of lncRNA and mRNA indicated positive correlation. Protein phosphatase 1, regulatory (inhibitor) subunit 1B (*Ppp1r1b*)^24^ or versican (*Vcan*)^25^ showed gradually downregulation or upregulation from P1 to P28, opposite to the expression patterns of their neighboring lncRNAs uc007lgb.1 and uc007rjm.1, respectively (Fig. 3c, left and middle panel). In addition, ENSMUST00000127169 was rapidly increased from P1 to P7 but gradually declined from P7 to P28, opposite to its neighboring gene proteolysis of human protein tyrosine kinase 7 (*Ptk7*)^26^ (Fig. 3c, right panel). These data suggested a potential negative regulation. Nuclear lncRNAs usually transcriptionally or epigenetically regulate gene expression^18^, while lncRNAs in cytosol are more likely to modulate gene expression post-transcriptionally^15^. Therefore, we also determined the nuclear or cytosolic distribution of these 6 lncRNAs presented in Fig. 3b,c. Data showed that uc008ffq.1 was mainly located in nucleus, indicating its potency to transcriptionally regulate *Ccbe1*. Meanwhile, the other 5 lncRNAs, located equally in nucleus and cytoplasm, may modulate their neighboring genes either transcriptionally or post-transcriptionally (Supplementary Fig. S1).

### Gene Ontology (GO) and pathway analysis

**Figure 4:**
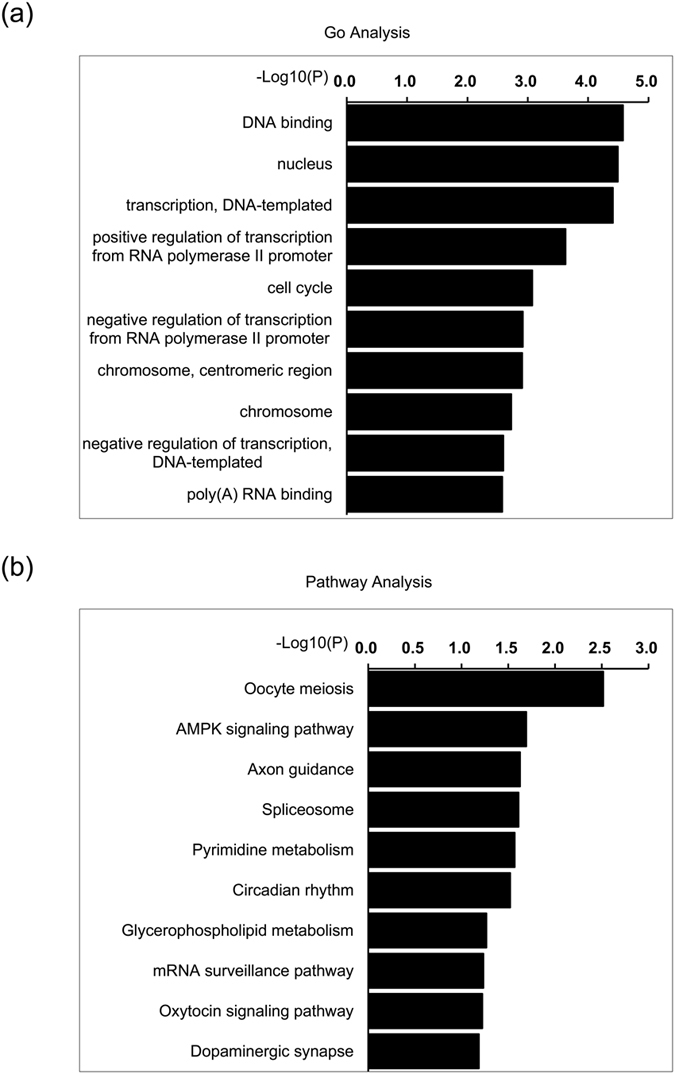
GO and pathway analysis of differentially expressed neighboring coding genes in neonatal mouse heart. (**a**,**b**) GO (**a**) and pathway (**b**) analysis of the neighboring coding genes corresponding to differentially expressed lncRNAs. The vertical and horizontal axes represent the biological process/pathways and −Log10 (*p* value) of the corresponding biological process/pathways, respectively. The top 10 significant involved GO items and pathways (*p* < 0.001) are shown. Fisher exact test for (**a** and **b)**.

GO analysis of the neighboring coding genes related to the 1,146 differentially expressed lncRNAs showed the significantly changed GO terms for the neighboring gene expression patterns were mainly involved in DNA synthesis-associated process, such as DNA binding, DNA-templated transcription and cell cycle (Fig. 4a), which might be important for cardiomyocyte proliferation. Pathway analysis identifying the enriched pathways for the neighboring coding genes showed that some of them were involved in development and metabolism-associated pathways (Fig. 4b), such as AMP-activated protein kinase (AMPK)^27^.

### Microarray validation by qRT-PCR

**Figure 5:**
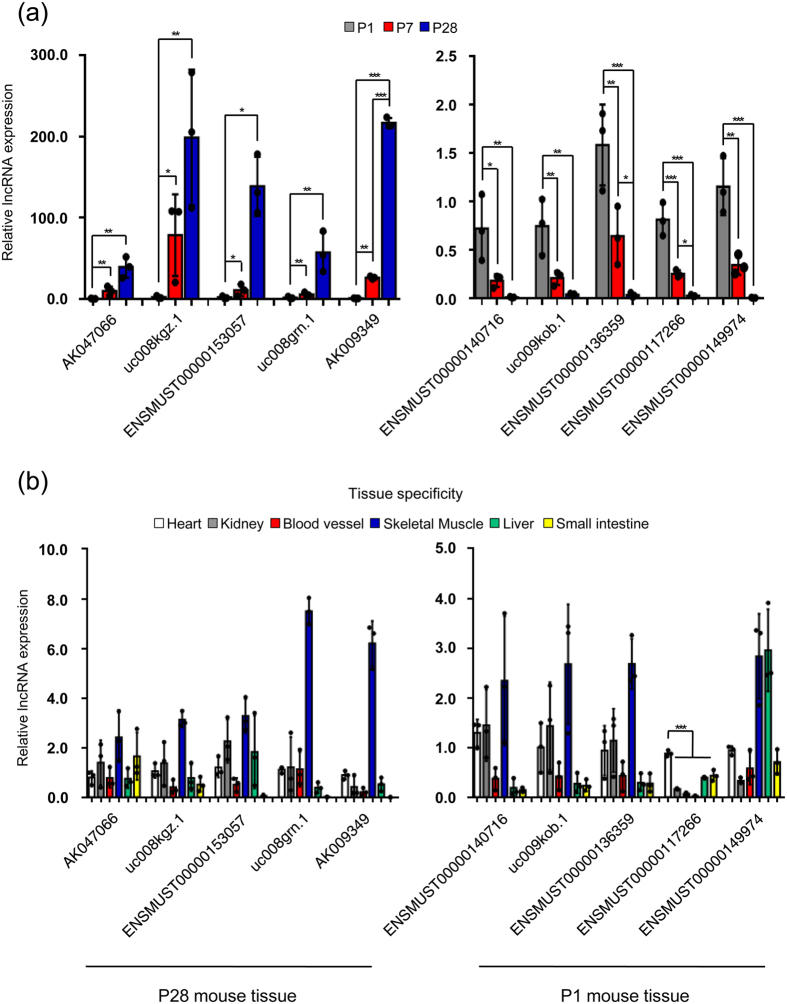
Validation of lncRNA expression in microarray data by real time qRT-PCR. (**a**) The expression levels of 5 consistently upregulated lncRNAs (left panel) and 5 consistently downregulated lncRNAs (right panel) during neonatal mouse heart development were validated by real time qRT-PCR. (**b**) The expression levels of the above-validated lncRNAs in different mouse tissues that contain smooth muscle cells were determined by real time qRT-PCR. Data are normalized to *Gapdh*. Data are shown as mean ± S.D. *, ** and ***indicate *p* < 0.05, *p* < 0.01 and *p* < 0.001, respectively, which denote statistical comparison between the two marked groups. One-way ANOVA for (**a)** (*post hoc*: LSD multiple comparison test for AK047066, uc008kgz.1, uc008grn.1, ENSMUST00000140716, uc009kob.1, ENSMUST00000136359, ENSMUST00000117266 and ENSMUST00000149974; Dunnett T3 multiple comparison test for ENSMUST00000153057 and AK009349) and (**b)** (*post hoc*: LSD multiple comparison test). (n = 3 samples per time point).

To validate the microarray data, we chose 5 most consistently upregulated and 5 most consistently downregulated lncRNAs from P1 to P28 with the highest fold changes, respectively, and analyzed their expression levels in P1, P7 and P28 mouse hearts by qRT-PCR (Fig. 5a and Supplementary Fig. S2). The differential gene expression levels by qRT-PCR was in agreement with that found in the microarray data sets. From the array data, we found three differentially expressed neighboring genes, *Ppp1r1c* (associated with uc008kgz.1), *AW112010* (associated with uc008grn.1) and SAP30-like (*Sap30l*) (associated with ENSMUST00000117266), showed consistent expression patterns with their neighboring lncRNAs among these 10 lncRNAs (Data not shown), which were also validated by qRT-PCR (Supplementary Fig. S3).

As many lncRNAs had specific tissue expression pattern^19^, we next explored whether the above-validated lncRNAs showed a cardiac specificity. We analyzed heart, kidney, blood vessel, skeletal muscle, liver and small intestine to determine the cardiac-enriched lncRNAs *via* qRT-PCR. The age of mouse chosen for lncRNA tissue specificity was based on the higher cardiac expression level of associated lncRNAs at P1 or P28. Subsequently, we identified one novel lncRNA, ENSMUST00000117266, highly expressed in the hearts as compared to other tissues (Fig. 5b). The data was acquired and normalized by using *18S* and *Gapdh* as internal controls (Supplementary Fig. S4).

### Functional analysis of ENSMUST00000117266

**Figure 6:**
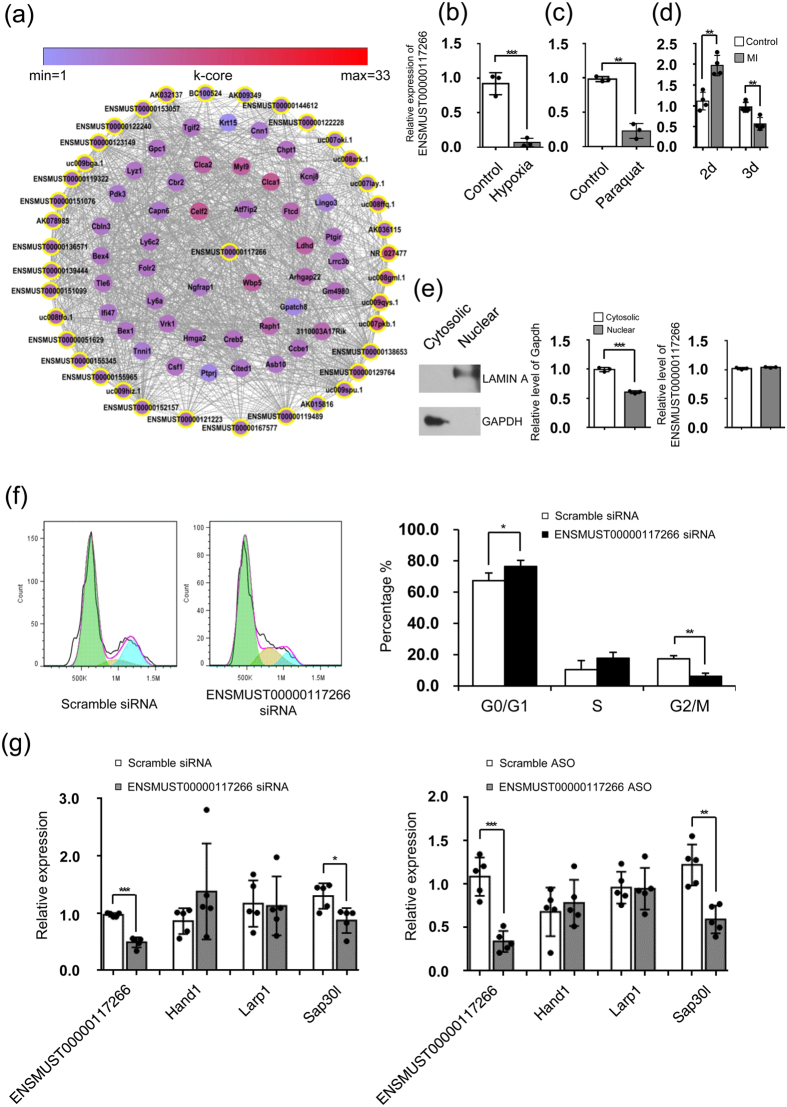
Gene co-expression subnetwork of ENSMUST00000117266 and its role in cardiomyocyte proliferation. (**a**) Co-expression subnetwork of ENSMUST00000117266. (**b**) Cardiomyocytes were cultured under normoxic or hypoxic conditions for 6 hours and the expression of ENSMUST00000117266 was determined by real time qRT-PCR. (**c**) 0.9% saline solution or paraquat (5 mg/kg) was daily administrated subcutaneously to neonatal mouse for 3 days after birth and the expression of ENSMUST00000117266 was determined in heart harvested at P3. (**d**) The expression of ENSMUST00000117266 at indicated time point after sham or MI surgery was determined by qRT-PCR. (**e**) The protein expression of LAMIN A and GAPDH in nucleus and cytoplasm of P1 mouse cardiomyocytes was analyzed by immunoblotting. The RNA levels of *Gapdh* and ENSMUST00000117266 in nucleus and cytoplasm of P1 mouse cardiomyocytes were analyzed by qRT-PCR. (**f**) Cell cycle analysis of primary mouse cardiomyocytes after ENSMUST00000117266 knockdown by using PI staining. Representative histograms and statistical data are shown. (**g**) The mRNA expressions of *Hand1, Larp1* and *Sap30l* in primary mouse cardiomyocytes were assessed by qRT-PCR after 48-hours ENSMUST00000117266 siRNA (left) or ASO (right) transfection. Data are normalized to *Gapdh*. Data are shown as mean ± S.D. *p < 0.05, **p < 0.01 and ***p < 0.001 denote statistical comparison between the two marked groups, respectively. *T* approximation test for (**b)** unpaired two-tailed Student’ *t*-test for (**d**,**f** and **g)**; paired two-tailed Student’ *t*-test for (**c** and **e)**. (n = 3 cultures per group in (**b** and **e)** n = 3 samples per group in (**c)**; n = 4 samples per group in (**d)**; n = 4 cultures per group in (**f**); n = 5 cultures per group in (**g**).

ENSMUST00000117266 is located at the reverse strand of chromosome 11 and stretches from 57,948,729 to 57,949,085. To explore the potential biological role of ENSMUST00000117266, we conducted signaling network analysis which provided the potential information for co-expressed genes of ENSMUST00000117266 (Fig. 6a). A total of 81 genes were involved in the subnetwork of ENSMUST00000117266, among which 44 were coding genes (Supplementary Table S7). In this subnetwork, high mobility group AT-hook 2 (*Hmga2*) was involved in cardiogenesis and normal cardiac development^28^, while *Ccbe1* was associated with cardiac and lymphatic progenitor lineage specification at early developmental stage^29^. It was noteworthy that Cbp/p300-interacting transactivator with Glu/Asp-rich carboxy-terminal domain 1 (*Cited1*), a transcriptional cofactor participating in embryonic development and embryo viability, was also involved in this subnetwork^30^. These data indicated a potential role of ENSMUST00000117266 in neonatal heart development.

To explore whether the expression level of ENSMUST00000117266 could be affected by cellular stress, we first applied an *in vitro* model of cardiomyocyte exposure to hypoxia to recapitulate reduced cardiomyocyte proliferation and increased cellular size^31^. ENSMUST00000117266 expression showed a significant downregulation in cardiomyocytes after 6-hour exposure to hypoxia (Fig. 6b). We also confirmed this finding by using an *in vivo* model by stimulating neonatal mice with paraquat, a reactive oxygen species (ROS) generator which is reported to induce the shift from hyperplastic-to-hypertrophic growth in neonatal heart (Fig. 6c)^3^. In addition, we also determined the expression of ENSMUST00000117266 in a mouse ischemic cardiac disease model. Surprisingly, ENSMUST00000117266 was significantly upregulated after 2 days (d) but soon downregulated after 3 d of myocardial infarction (MI) surgery (Fig. 6d)^32^. This finding suggested that ENSMUST00000117266 participated in the early adaptive process responding to the deleterious stress in ischemic cardiac diseases.

qRT-PCR analysis of subcellular distribution of ENSMUST00000117266 in neonatal mouse cardiomyocytes showed that ENSMUST00000117266 equally located in both nucleus and cytoplasm (Fig. 6e). To further determine the role of ENSMUST00000117266 in the proliferation of cardiomyocytes, we utilized the siRNA strategy to knockdown ENSMUST00000117266. We found the percentage of cardiomyocytes in G0/G1 phase of cell cycle was significantly increased but the percentage of cardiomyocytes in G2/M phase was significantly decreased (Fig. 6f), suggesting that ENSMUST00000117266 was involved in regulating cardiomyocyte proliferative activity. In addition, the mRNA expression level of a neighboring coding gene of ENSMUST00000117266, *Sap30l*, which was previously shown to regulate hemoglobin synthesis and erythropoiesis in early development^33^, was also significantly downregulated (Fig. 6g, left panel). However, the expression of two other neighboring genes, heart and neural crest derivatives expressed transcript 1 (*Hand1*) and La ribonucleo protein domain family member 1 (*Larp1*), was almost unchanged after ENSMUST00000117266 knockdown. To further confirm our conclusion, we also utilized antisense oligonucleotides (ASOs) method, which targets both pre-mRNA and mature mRNA^34^. Consistently, we also observed decreased mRNA level of *Sap30l* after ENSMUST00000117266 knockdown (Fig. 6g, right panel). These results suggested that ENSMUST00000117266 was likely to function as a regulator of cardiomyocyte proliferative activity *via* its neighboring coding gene *Sap30l*.

## Discussion

Heart development is a multi-factorial and complex process involving many signaling pathways that are regulated by growth factors, cytokines, and cardiogenic transcription factors. Non-coding genes constitute the majority of human genome while coding genes only account for 1.2%^35^. Previously, several elegant studies have identified cardiac-enriched lncRNAs in cardiovascular lineage commitment^12^, embryonic heart development^18^,^36^,^37^,^38^,^39^, and cardiovascular diseases such as cardiac hypertrophy and heart failure^16^,^40^. Our current study is focused on the expression profiles of lncRNAs in the hearts during the early postnatal growth stage, which is associated with the critical transition from hyperplastic to hypertrophic growth with dynamic cellular processes orchestrated by a complex signal network^1^,^2^,^3^. Characterizing the lncRNA expression profiles during this transition offers us a new horizon to better understand the complex regulatory network for heart development and maturation. The lncRNA expression profile in the early postnatal hearts also bridges between fetal and adult heart physiology.

Previous studies identified 3,997 differentially expressed lncRNAs (fold change ≥2.0) between embryonic and adult mouse heart^37^. The present study observed total 1,146 differentially expressed lncRNAs (fold change ≥2.0) in mouse hearts at three time points P1, P7 and P28, which covered the period from neonatal age to post lactation stage. Even if the fold change between P1 and P28 was set to over 10.0, there were still 153 significantly changed lncRNAs, 10 of which were found to be differentially expressed between embryonic and adult mouse heart^37^. These highly dynamic changes of lncRNA expressions during the early postnatal stage suggested their crucial roles in cardiac adaptation response after the birth, such as to the oxygenation state, neuroendocrine stimulation^41^, changes in hemodynamics^42^, and lactation^3^,^43^. GO and pathway analysis of neighboring genes showed that the major biological processes and pathways were predominantly associated with cardiac development and growth, including cell cycle regulation, DNA-templated transcription, as well as AMPK signaling pathway as previously reported^27^ and also in consistent with recent studies that lncRNAs in developing embryos were mainly involved in cell cycle regulation, cell differentiation, and cell fate commitment^38^. Furthermore, our STC analysis showed that lncRNAs in Profile #9 and #4 were persistently downregulated from P1 to P28, suggesting that these transcripts might mainly act on the embryonic heart development. LncRNAs in Profile #11, #14 and #1 were expressed with the highest level at P28, which supported their roles in maintaining adult heart function. Interestingly, our data also revealed 44.6% of total differentially expressed lncRNAs that were maintained higher expression levels at P1 and P7 but dramatically down-regulated to a near undetected level at P28. We speculated that these lncRNAs might play a more specific role in the period of early postnatal heart maturation and likely mediating the transition of cardiac hyperplastic growth to hypertrophic growth.

Several lncRNAs are involved in heart development and function by regulating neighboring genes, such as the pair of Fendr-*Foxf1* and Kcnq1ot1-*Kcnq*1^14^,^18^. Our data also showed numerous lncRNA-mRNA co-expressed pairs either in positive or negative manner. The positive pair of uc008ffq.1-*Ccbe1* might be involved in early cardiac development *via* modulating migration and proliferation of cardiac precursors cells^29^,^44^. Another positive pair, uc008kgz.1-*Ppp1r1c*, might be involved in maintaining normal function of cardiomyocytes^22^. The rapid expression change of ENSMUST00000127169 from P1 to P7 and P7 to P28 suggests its potential role in early heart development through negatively regulating the mRNA expression of *Ptk7*^26^. Additionally, we also found a portion of overlapping sequences existed in two pairs of lncRNA-mRNA partners (ENSMUST00000127169-*Ptk7* and uc008ffq.1-*Ccbe1*), which further suggests the potential interactions between the lncRNAs and their corresponding neighboring coding genes. In our present study, we also found several lncRNAs that were previously reported to be involved in heart development and function might also participate in the regulation of postnatal heart maturation. For example, Bvht (AK143260), known as a lncRNA inducing cardiac lineage commitment during embryonic period^12^, was upregulated more than 2.0-fold in P28 mouse heart compared to that in P1. This suggested that Bvht might also be involved in the fetal-to-adult physiology switching in the early postnatal hearts. Another well-known lncRNA, H19 (ENSMUST00000149974), has been previously identified as a typical transcript participating in cardiac hypertrophy and heart failure^40^,^45^. We found in our current study that H19 was gradually downregulated from P1 to P28, suggesting that H19 downregulation could be also involved in the transition.

Our study also revealed one previously unreported cardiac-enriched lncRNA, ENSMUST00000117266, with higher expression at P1 and downregulated at P28. Co-expression network analysis suggests its potential role in the regulation of cardiac development. Hypoxia *in vitro*, paraquat (ROS inducer) and MI *in vivo* altered ENSMUST00000117266 expression further supports the above hypothesis. Furthermore, siRNA knockdown of ENSMUST00000117266 confirmed its role in promoting G0/G1 to G2/M phase transition in cardiomyocytes, suggesting ENSMUST00000117266’s role in regulating cardiomyocyte proliferative activity^46^. Further analyses of the role of ENSMUST00000117266 in developing hearts and postnatal hearts will be performed in both loss-of-function and gain-of-function mouse models. Additional biochemical analyses will be needed to validate our bioinformatic data. Nevertheless, our study provided an important information of the dynamic changes of lncRNA in early postnatal hearts.

In conclusion, our study identified differentially expressed lncRNAs in hearts during the early postnatal stage. STC, GO and pathway analysis provided lncRNA clusters that are associated with heart transition from hyperplastc to hypertrophic growth. The cardiac-enriched intergenic lncRNA ENSMUST00000117266 is likely involved in regulating hyperplastic growth during development. These profiling data helps us to better understand the role of lncRNAs during postnatal heart growth and maturation and provides novel candidate targets for potential use in regenerative cardiology in the future.

## Materials and Methods

### Experimental animals

C57BL/6 J mice were purchased from the Experimental Animal Center of the Third Military Medical University (Chongqing, China). The mice were housed in the following conditions: 12-hour light/dark cycle, 22–25 °C, periodic air changes and free access to water and food. All experiments performed in this study were approved by the local animal ethics committee at Third Military Medical University in accordance with the principles published in the National Institutes of Health Guide for the Care and Use of Laboratory Animals (NIH Publication No. 85–23, 1996, revised 2011; available from: www.nap.edu/catalog/5140.html). Mice used in this work were anaesthetized by 7% chloral hydrate (350 mg/kg body weight), followed by decapitation, and then hearts were quickly removed from the sacrificed mice.

### Microarray analysis

Triplicate P1, P7 and P28 mouse hearts acquired from 3 different littermates (Pup for each time point in one littermate was randomly selected) were used for profiling both lncRNAs and coding mRNAs by using Arraystar Mouse LncRNA Microarray v2.0 (Agilent Technologies). We took the mouse hearts at P28 (1 week after lactation), to reduce the influence of lactation on cardiomyocyte proliferation^43^, instead of using P21 as reported previously^5^. Total RNA was prepared using Trizol^®^ Reagent (Life Technologies). By using random hexamer primers, RNA was amplified and transcribed into fluorescent complementary RNA (cRNA) along with the whole length of transcripts without 3′ bias. After purification with RNeasy Mini Kit (Qiagen), the labeled cRNAs were hybridized on the LncRNA Microarray v 2.0 (8 × 60 K). Microarray was then washed and scanned by Agilent Microarray Scanner (Agilent Technologies, p/n G2565BA). Agilent Feature Extraction software (Agilent Technologies) was utilized to read and analyze the data. Quantile normalization and subsequent data processing were conducted by using the GeneSpring GX v11.5.1 software package (Agilent Technologies). After quantile normalization of the raw data, lncRNAs and mRNAs, which meet the requirement that at least 3 out of 12 samples have flags in Present or Marginal (“All Targets Value”), were chosen for further data analysis. By using lncRNA microarray which has probes for 31,423 lncRNAs totally, 18,158 lncRNAs were detected in all samples, while the others showed too low signal intensity to be detected. The gene expression data was then analyzed by the random variance model^47^. LncRNAs and mRNAs, with statistical significance among the three groups, were identified by FDR value less than 0.05. Volcano plot filtering and hierarchical clustering were used to characterize the lncRNA profiles with fold change ≥2.0 and FDR <0.05. Among the 18,158 lncRNAs, a one-way ANOVA was performed between the three indicated time points to identify differentially expressed lncRNAs and mRNAs, which were defined as more than 2.0-fold changes between P1, P7 and P28. We divided the differentially expressed lncRNAs into 4 categories: P7 vs. P1 up, P28 vs. P7 up; P7 vs. P1 down, P28 vs. P7 down; P7 vs. P1 up, P28 vs. P7 down; P7 vs. P1 down, P28 vs. P7 up and showed them in hierarchical clustering maps by using the Agilent GeneSpring GX software (Agilent Technologies).

### STC, hierarchical clustering, GO and pathway analysis of differentially expressed lncRNAs and neighboring mRNAs

STC algorithm of gene expression dynamics was utilized to profile the gene expression time series and identify the most distinct gene clusters at indicated time points^48^. Due to the different expression tendencies of the differentially expressed lncRNAs and mRNAs at different time points, we established 16 unique expression profiles for clustering gene expression of short time-series by utilizing the clustering algorithm, which assigned each gene to the mostly matched expression profile model according to the correlation coefficient. The clustering algorithm and standard hypothesis test were used to classify and identify the profiles that have more significant genes assigned.

To further explore whether the expression of neighboring gene transcripts were correlated with the relative expression of lncRNAs, we characterized 59 differentially expressed lncRNAs from 4 categories and their corresponding neighboring coding genes and presented them by heat map as described in microarray analysis part.

GO and pathway analysis based on Kyoto Encyclopedia of Genes and Genomes database were utilized to analyze the enrichment of these coding genes located nearby to the differentially expressed lncRNAs in GO items and biological signal pathways. Each neighboring gene located nearest to its corresponding lncRNA with 300 kilobase (kb) was selected.

### Real time qRT-PCR

Total RNA was prepared using Trizol^®^ agent (Life Technologies) according to the manufacturer’s instruction by using heart tissue or cardiomyocytes from C57BL/6 J mouse. The cDNA was synthesized from 1 μg total RNA by using Bestar™ qPCR RT Kit (DBI Bioscience). Real time qRT-PCR was conducted by using 2× SyberGreen mixture (DBI Bioscience) on ABI Prism 7700 Sequence Detector (Applied Biosystems). *Gapdh* or *18S* was taken as a housekeeping gene to normalize gene expression by using the classical ^ΔΔ^Ct method. Primers used for each gene were listed in Table 1.

### Construction of ENSMUST00000117266 co-expression subnetwork

We conducted the gene co-expression subnetwork of ENSMUST00000117266 and genes that have higher interaction coefficient with it as published literature described^49^. The threshold value of Pearson correlation was set to 0.97 to ensure a better visual representation. Gene pairs in the subnetwork were measured by the significance of correlation test. K-core value was used to separate the large amount of genes into smaller subnetworks, which reflects the relationship among the associated genes. Cycle nodes represent genes. Edge linking two nodes means the interaction type between two genes. Cycle nodes with different colors represent different k-core values, which mean the power of the gene in the co-expression subnetwork. The degree of a gene is defined as the number of other genes that interact with it, which is shown by the size of its cycle node.

### Isolation, culture and treatment of cardiomyocytes

Neonatal mouse cardiomyocyte culture was prepared as described in the literature^50^. Briefly, hearts from P1 C57BL/6 J mice were excised and ventricles were washed, minced (1 mm^3^) and digested with 0.25% trypsin (Beyotime Institute of Biotechnology). After digestion was stopped by complete medium (Dulbecco’s modified Eagle’s medium supplemented with 10% fetal bovine serum (Life Technologies)), cells were collected by centrifugation and then cultured in complete medium with 0.1 mM 5-bromo-2-deoxyuridine (Sigma) at 37 °C at the density of 70,000 cells/cm^2^ in humidified 5% CO_2_ atmosphere for further use. After 48 hours of culture in the complete medium, cardiomyocytes were incubated in normoxia (21% O_2_, 5% CO_2_) or hypoxia (1% O_2_, 5% CO_2_) in a hypoxia chamber (Thermo Fisher Scientific) for 6 hours at 37 °C.

### Transfection with siRNAs or ASOs

We performed siRNA transfection using X-tremeGENE siRNA transfection kit according to the manufacturer’s protocol (Roche). Briefly, neonatal mouse cardiomyoyctes were transfected with the mixture of three different target-specific ENSMUST00000117266 siRNAs (GenePharma) and 10 μl X-tremeGENE siRNA transfection reagent. For ASO transfection, neonatal mouse cardiomyoyctes were transfected with the mixture of ENSMUST00000117266 ASOs (Gene Pharma) modified with phosphorothioates and locked nucleic acid using lipofectamine 2000 according to the manufacturer’s protocol (Invitrogen). Scramble siRNA or ASO was used as a nonspecific control. After 8 hours of transfection, cells were refreshed with the complete medium and cultured for another 48 hours before harvesting for RNA extraction. The sequences of siRNAs and ASOs were shown in Table 2.

### Neonatal mouse treatment

C57BL/6 J neonatal mice from one littermate were randomly divided into 2 groups equally, and were treated with daily paraquat (5 mg/kg, Sigma) or 0.9% saline, respectively, through subcutaneous injection from P1 to P3, as described in the ref. 3. Neonatal mouse subcutaneously injected 0.9% normal saline for the same time served as control group. 12 hours after the last injection of paraquat or normal saline, mice were sacrificed and hearts were harvest for further real time qRT-PCR.

### MI

For sham and MI surgery, 12-week-old C57BL/6 J mice were randomly divided into the two groups equally. Mice were treated with permanent ligation of the left anterior descending coronary artery or a sham operation^51^.

### Nuclear and cytosolic RNA extraction

Nuclear and cytosolic RNA extractions were performed according to the manufacture’s protocol (PARISTM Kit, Life Technologies). Briefly, primary mouse cardiomyocytes were washed once with PBS, spun down and resuspended in 300 μL ice-cold cell fractionation buffer. After incubation on ice for 10 min and centrifuged at 500 × *g*, nuclear and cytosolic fractions were obtained from the supernatant and pellet, respectively. Then nuclear pellets were further lysed in cell disruption buffer. Nuclear and cytosolic lysates were mixed with equal volume of 2× lysis/binding solution and 100% ethanol. Next, the mixtures were applied to the filter cartridge and washed by wash solution. Finally, nuclear and cytosolic RNAs were eluted by elution buffer.

### Immunoblotting

Nuclear and cytosolic extraction of P1 mouse cardiomyocytes were lysed with RIPA buffer (Beyotime) and subjected to immunoblot analysis as described before^52^,^53^,^54^. Antibodies against LAMIN A and GAPDH were purchased from Abcam and Cell Signaling, respectively.

### Cell cycle analysis

Cardiomyocytes were washed twice with ice-cold PBS and fixed in 75% ice-cold ethanol overnight at 4 °C. Then, the fixed cardiomyocytes were stained with 50 mg/ml propidium iodide (PI) containing 50 mg/ml RNase A (DNase free) for 30 min at room temperature with protection from light. Data were analyzed using Accuri C6 flow cytometry (BD Biosciences) and Flow Jo software, as described previously^55^.

### Statistical analysis

Unpaired or paired Student’ *t*-test was used to compare two independent or dependent groups, respectively, if they are normally distributed. The *T* approximation test was used if they are with unequal-variance. One-way ANOVA was carried out with an appropriate *post hoc* test for comparison of multiple groups. Fisher’s exact test was used in STC, GO and pathway analysis. Generalized linear models were used in the randomized block design with litters as the block factor. All tests were two-sided. A *p* < 0.05 was considered statistically significant. Data are presented as mean ± S.D.

## Additional Information

**How to cite this article**: Sun, X. *et al*. Profiling analysis of long non-coding RNAs in early postnatal mouse hearts. *Sci. Rep.* **7**, 43485; doi: 10.1038/srep43485 (2017).

**Publisher's note:** Springer Nature remains neutral with regard to jurisdictional claims in published maps and institutional affiliations.

## Supplementary Material

Supplementary Information

Supplementary Table S1

Supplementary Table S2

Supplementary Table S3

Supplementary Table S4

Supplementary Table S5

Supplementary Table S6

Supplementary Table S7

## Figures and Tables

**Table 1:** Primers used in real time qRT-PCR.

Seqname	Primer sequences (5′-3′)
uc008ffq.1_F	GTCATATTGCTGTGTGGAGCC
uc008ffq.1_R	TAACAATGACGGCCCTGGGT
uc008kgz.1_F	CTTCACCGCTCTGTCTTGGT
uc008kgz.1_R	CCTCTGGGGCAATCTGACTC
uc007lay.1_F	CCCGCAGACTTGGAGAAAGT
uc007lay.1_R	GTTTCGATGGCCTTCATCGC
uc007lgb.1_F	TGCCTGATGGCCTTTCTCAG
uc007lgb.1_R	CTTTCAGTGATGGGGGCGTA
uc007rjm.1_F	CCAGGAAGTCACACCCGTC
uc007rjm.1_R	CATTGAGGTACCGGCAGGAG
ENSMUST00000127169_F	TTAGGGCGCGGTTGTCTTCT
ENSMUST00000127169_R	TGGATTTGGGGCCAGTAGATG
AK047066_F	CCTTTCTCCGTGTGGTAGGG
AK047066_R	CCTTCTCCGGCTCCAAATGA
ENSMUST00000153057_F	GGACGCTTTCAACCAGAGGA
ENSMUST00000153057_R	ACGAGCTATACGCAGCTCAC
uc008grn.1_F	AGTCTTCTGCCATCAAGCCAA
uc008grn.1_R	ATCTCTCCTGAACGACCCAGT
AK009349_F	TGCTGTTGGCCGTTTAGTGA
AK009349_R	AGACGAGTTGTTCGGGGATG
ENSMUST00000140716_F	TGGAGTCCCGGAGATAGCTT
ENSMUST00000140716_R	GTTGCCCTCAGACGGAGATG
uc009kob.1_F	GCATCGCAAAGGCTGGAAAA
uc009kob.1_R	CTCCCCTTTATCCGACCAGC
ENSMUST00000136359_F	CACTGTATGCCCTAACCGCT
ENSMUST00000136359_R	CCCAACCTCCCTCCCTAGAA
ENSMUST00000117266_F	GGAAATGTCCGAAGGAAAGTCAG
ENSMUST00000117266_R	CCCTGCACTACAAATCTCCCAAC
ENSMUST00000149974_F	CCTCGCTCCACTGACCTTCTA
ENSMUST00000149974_R	CCTTTGCTAACTATCCTGCCTTT
*Ppp1r1c_*F	AGCACCTACTGTGCATGTCC
*Ppp1r1c_*R	TGGGCTGTTCCCCGTTTATT
*AW112010_*F	AGTCTTCTGCCATCAAGCCA
*AW112010_*R	ATCTCTCCTGAACGACCCAGT
*Sap30l*_F	CAAGAGCGTAAGGCACCTGT
*Sap30l*_R	TCAGGGATGTCAGCATCGTG
*Hand1*_F	ACATCGCCTACCTGATGGAC
*Hand1*_R	TAACTCCAGCGCCCAGACT
*Larp1*_F	ATTATGCCGACCTGTCTCCC
*Larp1*_R	CCCCACTGAGATCCTTTGGT
*Gapdh_*F	AGGTCGGTGTGAACGGATTTG
*Gapdh_*R	TGTAGACCATGTAGTTGAGGTCA
*18S*_F	CCGCCGCCATGTCTCTAGT
*18S*_R	CTTTCCTCAACACCACATGAGC

**Table 2:** Sequences of ENSMUST00000117266 siRNAs and ASOs.

siRNA	sequences (5′-3′)
siRNA1_F	GCGACUUGUGCCUAACUUUTT
siRNA1_R	AAAGUUAGGCACAAGUCGCTT
siRNA2_F	GCAUGUUGAUCGCAAUGAATT
siRNA2_R	UUCAUUGCGAUCAACAUGCTT
siRNA3_F	CCUGACAAUCAUUACGACUTT
siRNA3_R	AGUCGUAAUGAUUGUCAGGTT
ASO1	AUGCCUAUUAGGUAUGGCCTT
ASO2	UUCGGAAAUGCCUGUAAGGTT
