# Supplementary material for: Profiling analysis of long non-coding RNAs in early postnatal mouse hearts
Source: Sci Rep. 2017 Mar 7;7:43485. doi: 10.1038/srep43485 (PMC5339910; doi:10.1038/srep43485)
Supplement: Supplementary Information [file srep43485-s1.pdf]

# Profiling analysis of long non-coding RNAs in early postnatal mouse hearts

Xiongshan Sun,[1,2,*] Qi Han,[1,2,*] Hongqin Luo,[1,2] Xiaodong Pan,[1,2] Yan Ji,[1,2] Yao Yang,[1,2] Hanying Chen,[3] Fangjie Wang,[1,2] Wenjing Lai,[1,2] Xiao Guan,[1,2] Qi Zhang,[1,2] Yuan Tang,[1,2] Jianhong Chu,[4,5] Jianhua Yu,[4] Weinian Shou,[1,2,3] Youcai Deng,[1,2] Xiaohui Li[1,2]

## Supplementary Figures and Figure Legends

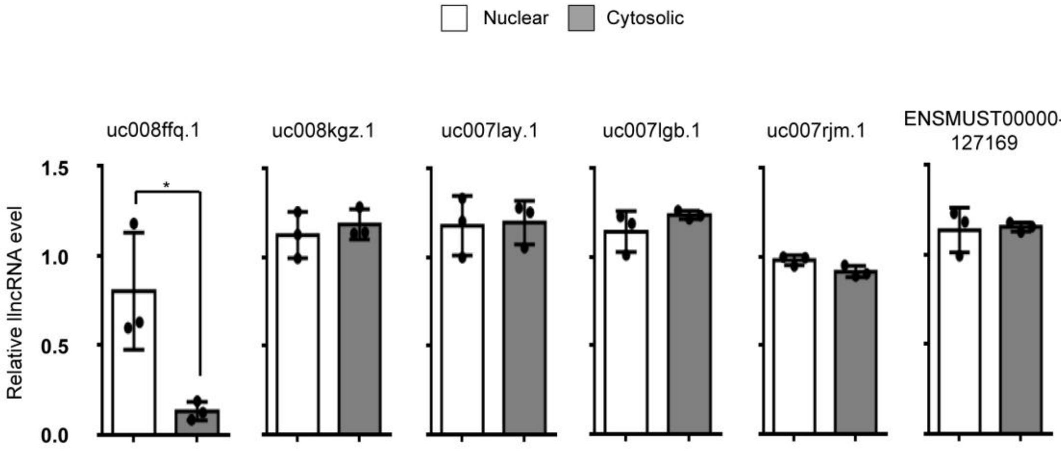


**Supplementary Figure S1. The cytosolic and nuclear distribution of lncRNAs determined by qRT-PCR analysis.** The RNA levels of lncRNAs in both cytoplasm and nucleus were determined *via* real time qRT-PCR. Data are presented as mean ± S.D. * indicates $p < 0.05$, which denote statistical comparison between the two marked groups. Paired two-tailed Student's *t*-test was used (n = 3 cultures per group).

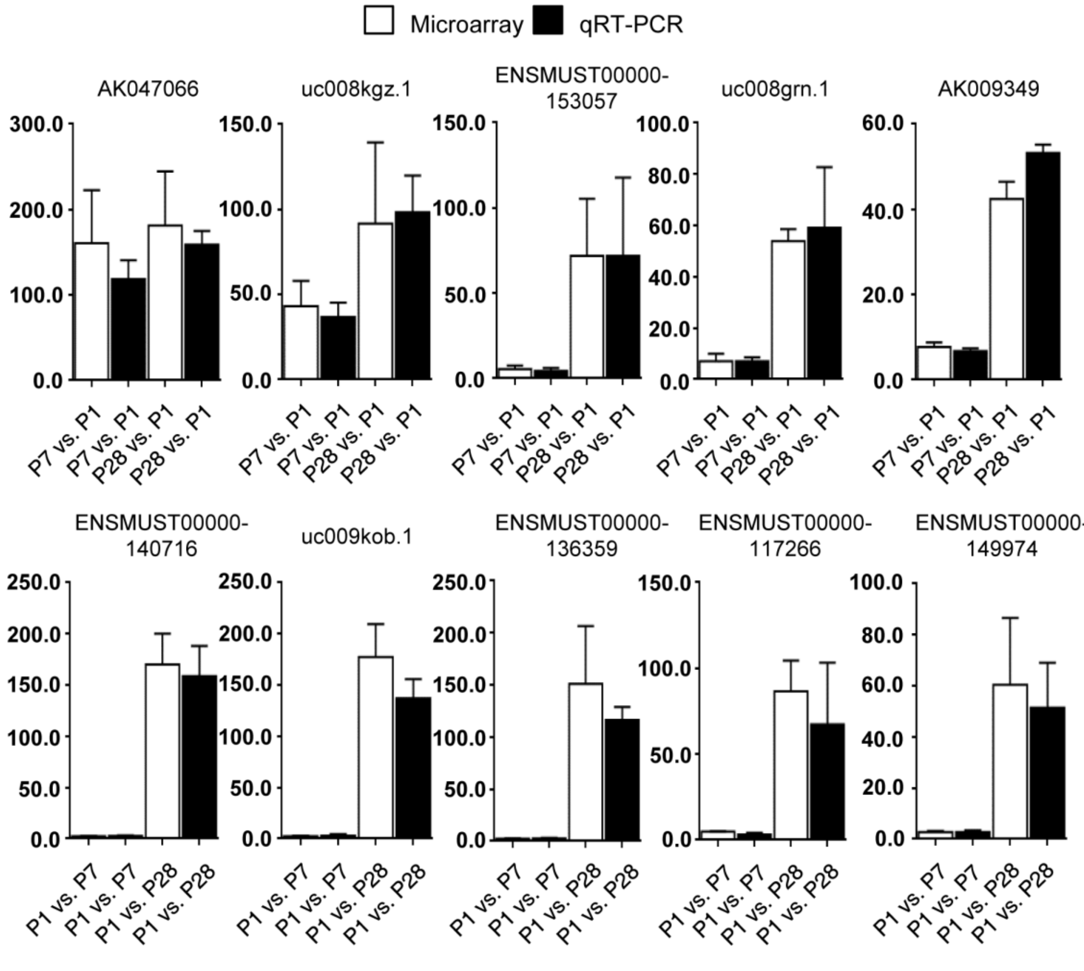


**Supplementary Figure S2. Microarray validation of the top 10 lncRNAs by real time qRT-PCR.** Fold changes between P1 and P7 or P1 and P28 of the top 10 lncRNAs are shown

for both microarray and qRT-PCR data, respectively. Data are presented as mean ± S.D.

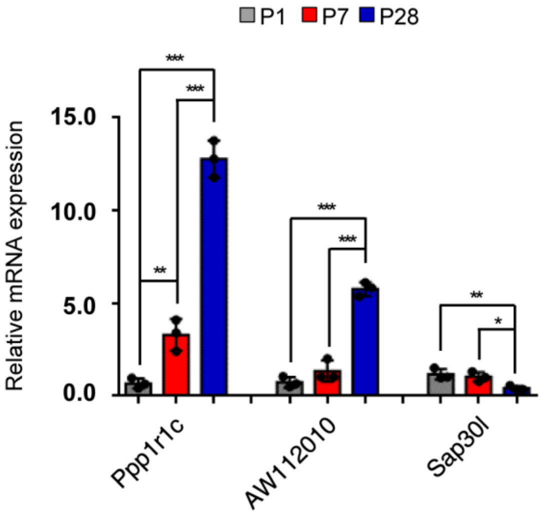


**Supplementary Figure S3. Microarray validation of the 3 differentially expressed neighboring genes by real time qRT-PCR.** The mRNA levels of *Ppp1r1c*, *AW112010* and *Sap30l*, related to lncRNA uc008kgz.1, uc008grn.1, and ENSMUST00000117266, respectively, in P1, P7 and P28 mouse heart were validated by real time qRT-PCR. Data are normalized to *18S*. Data are shown as mean ± S.D. *, ** and *** indicate $p < 0.05$, $p < 0.01$ and $p < 0.001$, respectively, which denote statistical comparison between the two marked groups. One-way ANOVA (*post hoc*: LSD multiple comparison test) was used.

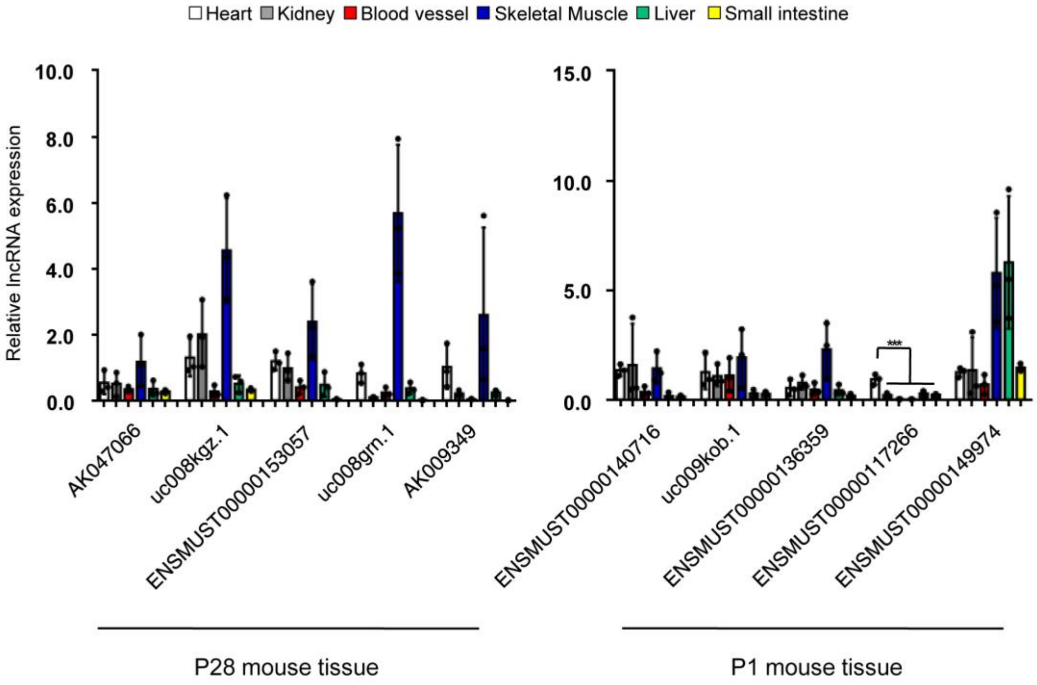


**Supplementary Figure S4. Tissue specificity validation of the top 10 lncRNAs by using *18S* as an internal control.** The expression levels of the top 10 lncRNAs in different mouse

tissues that contain smooth muscle cells were determined by real time qRT-PCR. Data were normalized to *18S*. Data are shown as mean ± S.D. *, ** and *** indicate $p < 0.05$, $p < 0.01$ and $p < 0.001$, respectively, which denote statistical comparison between the two marked groups. One-way ANOVA (*post hoc*: LSD multiple comparison test) was conducted. (n = 3 samples per time point).
